# Epidemiological and clinical implications of blood pressure measured in seated versus supine position

**DOI:** 10.1097/MD.0000000000011603

**Published:** 2018-08-03

**Authors:** Ernest Privšek, Margareta Hellgren, Lennart Råstam, Ulf Lindblad, Bledar Daka

**Affiliations:** a University Medical Centre Ljubljana, Ljubljana, Slovenia; b Institute of Medicine, Department of Public Health and Community Medicine/Primary Health Care, Sahlgrenska Academy, University of Gothenburg, Gothenburg; c Lund University, Department of Clinical Sciences in Malmö, Family and Community Medicine, Skåne University Hospital, Malmö, Sweden.

**Keywords:** blood pressure measurement, body position, clinical and epidemiological implications, hypertension, Vara–Skövde cohort

## Abstract

The evidence concerning how posture influences blood pressure is not consistent. The aim of this cross-sectional study was to consider the clinical and epidemiological implications of blood pressure measured in seated versus supine position, and to investigate the impact of age, sex, body mass index (BMI), and diabetes on these differences.

This study included 1298 individuals (mean age 58.6 ± 11.8 years) from the Vara-Skövde cohort at the 10 years’ follow-up visit in 2014. Physical examination included blood pressure measurements in seated and supine position. Self-reported information on diabetes status, hypertension, ongoing medication, leisure time physical activity, and smoking habits were obtained. Linear regression models accounted for differences in age, sex, BMI, and known diabetes.

Both systolic and diastolic blood pressure were significantly higher in the seated position [1.2 mm Hg, *P* < .001, 95% confidence interval (95% CI) 0.79–1.54 and 4.2 mm Hg, *P* < .001, 95% CI 4.08–4.71, respectively]. The prevalence of high blood pressure in seated position was higher (19.9%) than in supine position (13.5%). Linear regression analysis showed that age (β = −0.215, *P* < .001) and diabetes (β = −0.072, *P* = .012) were associated with smaller differences in postural diastolic blood pressure and BMI (β = 0.124, *P* < .001) with greater difference.

This study showed substantial postural differences in blood pressures measured in office. Measuring blood pressure in the supine position shows lower blood pressure readings when compared with the seated position. Clinicians should be aware of how age, BMI, and diabetes influence these differences.

## 1 Introduction

Blood pressure is one of the fundamental clinical measurements in medicine. It is also the basis for the diagnosis, management, treatment, and epidemiology of hypertension, and for research. Numerous factors affect the result of the measurement of blood pressure, varying from the technique and the selection of an accurate device to intrinsic variability of blood pressure and white-coat hypertension.^[1]^ The procedure itself influences the outcome of the measurement: the communication with the individual, patient education, attitude of observer, attitude of patient, arm circumference, cuff size, arm position, and patient posture. There is a general acceptance that posture affects the blood pressure. From supine to seated or standing position, the pressure usually drops.^[1]^ The 2013 European Society of Hypertension (ESH)/European Society of Cardiology (ESC) guidelines for the management of arterial hypertension recommend the mean of at least 2 blood pressure measurements in the seated position, spaced 1 to 2 minutes apart. The position of the arm, even in supine position, should be adjusted at heart level to avoid changes in blood pressure.^[2]^ However, there is still uncertainty regarding the importance of the postural differences in blood pressure measurements. In the 2003 ESH recommendations for blood pressure measurement, the authors state that the error made is unlikely to be significant in most people.^[1]^ On the contrary, several authors claim that there is a significant difference in blood pressures measured in seated and supine position,^[3–5]^ and therefore, they cannot be considered similar.^[2]^ In an overview from 2003, Netea et al^[6]^ point out that different studies have shown contradictory results and list the possible confounders that could explain these differences. Pickering et al^[7]^ state that it is widely accepted, that the diastolic blood pressure (DBP) when measured in a seated position is higher than when measured supine (by 5 mm Hg), although there is less agreement about the systolic blood pressure (SBP). Alternative body positions for measuring the blood pressure have also been proposed.^[3]^ Our review of the available publications on Medline after 2003 showed contradictory results (Table 1).^[3–5,8–11]^ Some of these studies contained one or more limitations regarding the study group or the methods used; however, they were all specifically designed for the purpose of determining the effect of body position on blood pressure. The aim of this cross-sectional study was to compare the blood pressure measurements in seated and supine position in a population-based cohort in Sweden, to investigate the clinical and epidemiological implications for the diagnosis of hypertension, and to elucidate the impact of age, sex, BMI, hypertension, and diabetes on these differences.

**Table 1:**
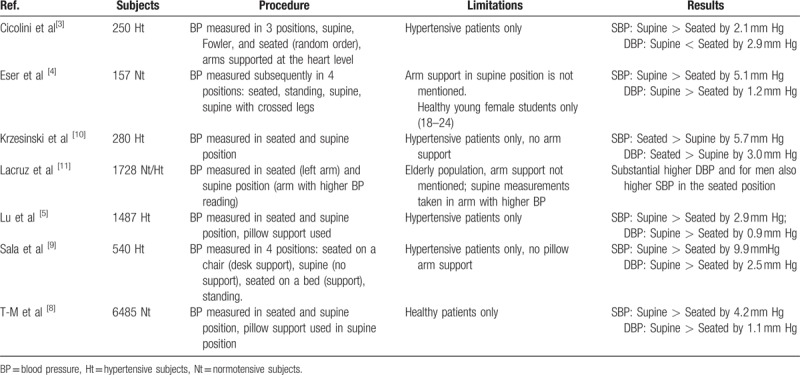
Overview of the existing publications regarding impact of the body position on blood pressure measurement.

## 2 Methods

### 2.1 Study design and subjects

**Figure 1:**
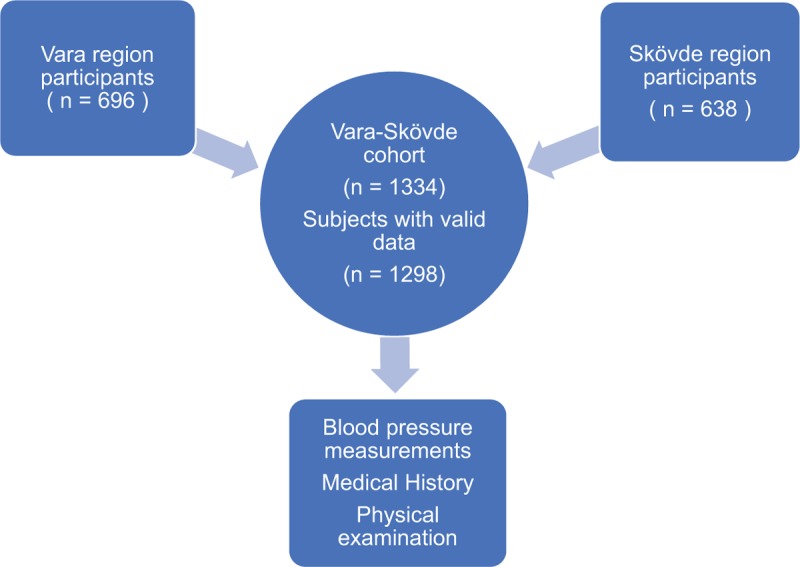
Study population.

This cross-sectional study investigated the Vara–Skövde cohort, which has been previously described.^[12]^ In brief, during 2002 to 2005, 2816 randomly selected participants (men N = 1400) were stratified by sex and 5-year age groups, from all individuals (no exclusion criteria) 30 to 74 years of age, with intentional oversampling (3-fold) in the age group 30 to <50 as compared with those aged ≥50 years. There were 1811 subjects who fulfilled all requirements for participation from the Vara population (81% participation rate) and 1005 subjects from the Skövde population (70% participation rate). During 2012 to 2014, a 10-years’ follow-up survey was completed. Within the Vara–Skövde cohort, 1954 subjects were consecutively invited to participate; of those, 85 died during the follow-up, and 35 subjects could not be reached because they had moved. The remaining 1834 subjects were invited to participate in the study. Of these, 490 individuals declined to participate, and 17 participants could not fulfil the study and were consequently excluded. In total, 1327 individuals (73% participation rate) were examined according to the study protocol.^[13]^ In 29 cases, complete data on blood pressure measurements were lacking, and these subjects were also excluded from further analyses. The final sample consisted of 1298 (men N = 638) subjects who had complete data including filling out the questionnaires, undergoing a physical examination, and having blood samples stored (Fig. 1). Only measurements from the follow-up visit were analyzed in this study. The Ethics Committee at the University of Gothenburg, Sweden, approved the study, and signed informed consent was obtained from all participants before participating in the study.

### 2.2 Measurements

**Figure 2:**
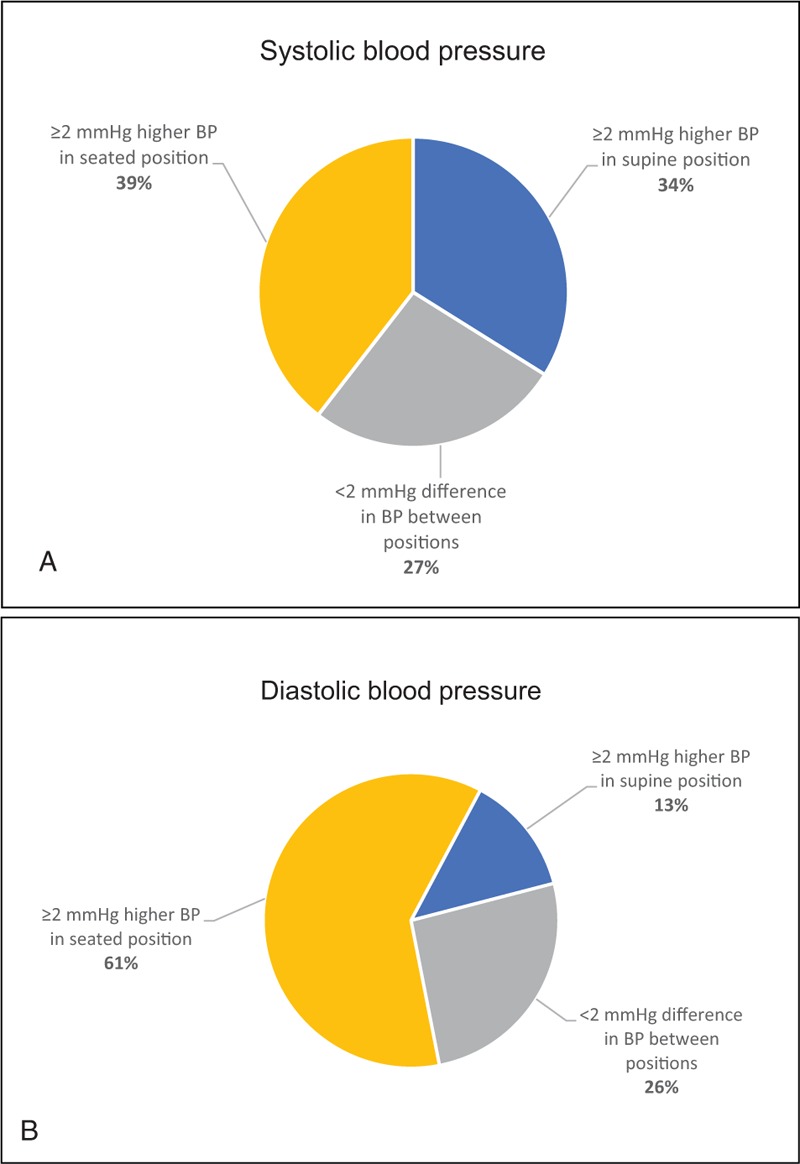
(A) Positional difference in systolic blood pressure (SBP). –(B) Positional difference in diastolic blood pressure (DBP).

Five specially trained nurses collected the information as previously described in detail.^[14]^ Participants completed validated questionnaires on medical history and lifestyles, including smoking habits and physical activity. Fasting blood samples were collected in all subjects and an oral glucose tolerance test (OGTT) was conducted on all subjects without known diabetes and type 2 diabetes was defined according to the WHO criteria.^[15]^ All ongoing medications were documented, and physical examinations included waist circumference, body weight (nearest 0.1 kg on a calibrated scale), body height (nearest cm), and heart rate. Blood pressures were measured in 2 positions, supine and seated, on the right arm, which was supported at the heart level using pillow or arm chair. All blood pressure measurements were done in accordance to ESH/ESC 2013 guidelines using Tricuff^[16]^ for automatic adjustment of cuff size to arm circumference. First, the patients took supine position, and after 5 minutes’ rest, 2 blood pressure measurements were taken with a 1-minute interval. Following that, noninvasive pulse wave measurements were conducted. Patients then took seated position and rested for 5 minutes before the 2 blood pressure measurements with 1-minute interval. On average, the time between the positions was 15 minutes. Mean values from 2 measurements were used in the study. Hypertension was diagnosed in accordance with national and international guidelines.^[17]^ The definition of hypertension was based on 3 consecutive high readings with 4-week intervals (≥140 systolic and/or ≥ 90 mm Hg diastolic in sitting position), or the participant had a known doctor's diagnosis with ongoing treatment. High blood pressure was defined as a 1-time measurement of SBP ≥140 mm Hg and/or DBP ≥ 90 mm Hg. BMI was estimated using the formula body weight (kg) divided by the square of body height in meters (m^2^). Obesity was defined as body mass index (BMI) more or equal 30 kg/m^2^ and overweight if BMI ≥ 25 but < 30 kg/m^2^.^[18]^ The positional differences in SBP and DBP (ΔSBP and ΔDBP) were calculated by subtracting supine blood pressure from the seated blood pressures, systolic and diastolic, respectively (ΔSBP = SBP_seated_ - SBP_supine_; ΔDBP = DBP_seated_ - DBP_supine_). Three categories were defined depending on the difference in blood pressures that the subjects had in different positions. The first category included subjects with considerably (≥ 2 mm Hg) higher blood pressures in supine position, the second category included subjects with small differences in blood pressure (< 2 mm Hg between positions), and the third category included subjects who had considerably (≥ 2 mm Hg) higher blood pressure in seated position (Fig. 2A, B).

### 2.3 Statistics

Data were expressed as means ± standard deviation (SD). Standard methods were used for descriptive statistics. The paired Student *t* test was used to compare the mean blood pressure values between the 2 postures. Linear regression models were used to assess interactions and to estimate the roles of possible confounders. All analyses were 2-sided, and *P* < .05 was considered statistically significant. Data were analyzed using IBM SPSS Statistics for Windows, Version 23.0. Armonk, NY: IBM Corp.

## 3 Results

### 3.1 Characteristics of the population

No significant differences in age or BMI were observed when comparing men and women; however, women had significantly lower SBP and DBP in both supine and seated positions and higher heart rate than men. The difference in ΔSBP and ΔDBP (positional difference in blood pressures) between sexes was small and not statistically significant. All comparisons between sexes were age adjusted (Table 2). Regarding SBP, most subjects (39%) had significantly higher blood pressure in seated position, one-third (34%) had significantly higher blood pressure in supine position, and 27% had small insignificant differences in blood pressure between the positions (Fig. 2A). Results of the DBP analysis were different – a majority (61%) of the subjects had considerably higher blood pressure in seated position, and while only 13% had considerably lower blood pressure in seated position, 26% of the subjects had smaller insignificant differences in blood pressures (Fig. 2B).

**Table 2:**
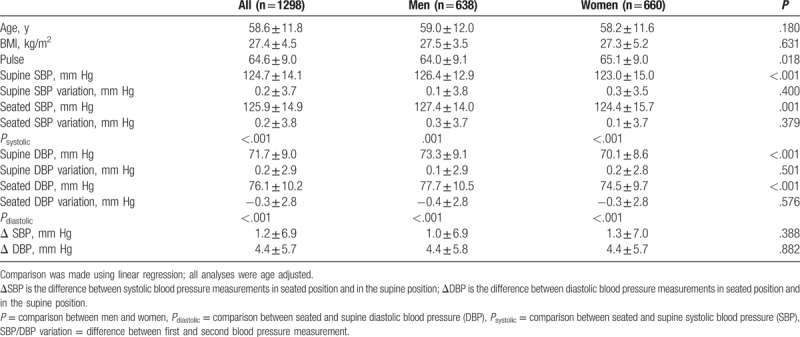
Characteristics of the study population.

### 3.2 Age group analysis

**Figure 3:**
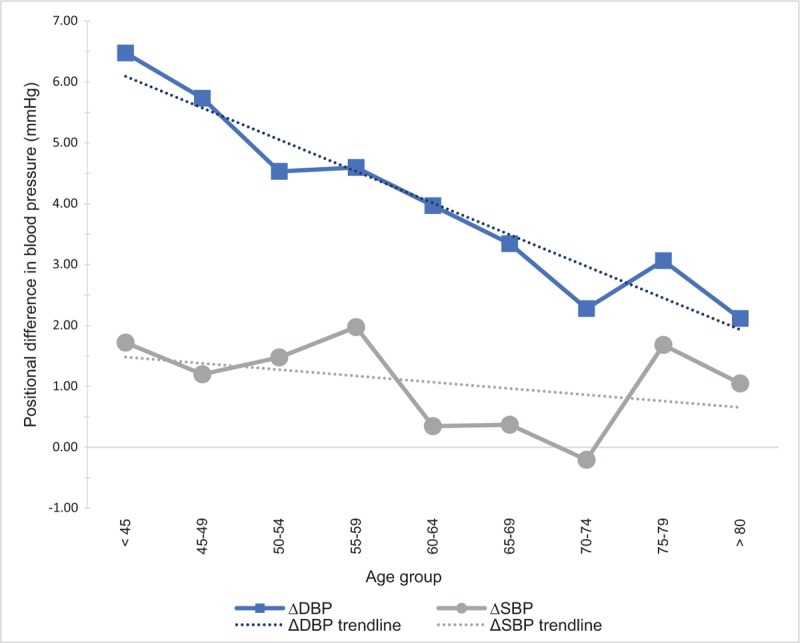
Mean positional difference in blood pressure in regard to age groups.

**Figure 4:**
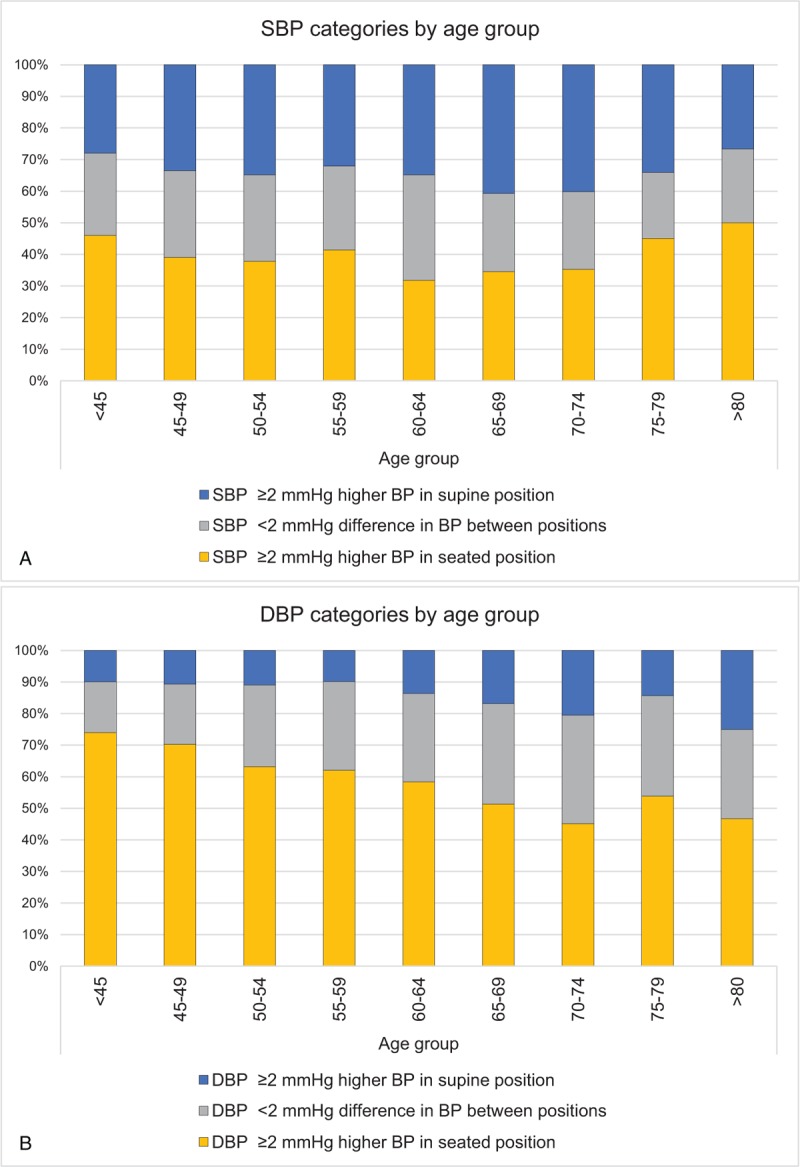
(A) Distribution of systolic blood pressure categories in regard to 5-year age group. (B) Distribution of diastolic blood pressure categories in regard to 5-year age group.

As summarized in Table 3, the mean SBP gradually increased across age groups from <45 to ≥80 years in both seated and supine position (*P* < .001). However, a gradual decrease in DBP with age was noted after 60 years of age (*P* < .001). The mean SBP was higher in the seated position in all age groups except for the age group 70 to 74 years. The greatest difference between positions was in the age group 55 to 59 years, where the average seated SBP was 2.0 mm Hg higher (*P* < .001). DBPs were significantly higher in the seated position in all age groups, and the biggest difference was in the age group < 45 years, wherein the mean seated diastolic pressure was 6.5 mm Hg higher. ΔSBP ranged from −23 to 33 mm Hg and ΔDBP ranged from −15 to 24 mm Hg. There was a significant difference in the mean ΔDBP between all the age groups (*P* < .001). A trend of gradual decrease in ΔDBP with age (Figs. 3 and 4) was observed and remained significant even after adjusting the analysis for known hypertension (β = −0.225, *P* < .001). The difference in mean ΔSBP between age groups was not statistically significant.

**Table 3:**
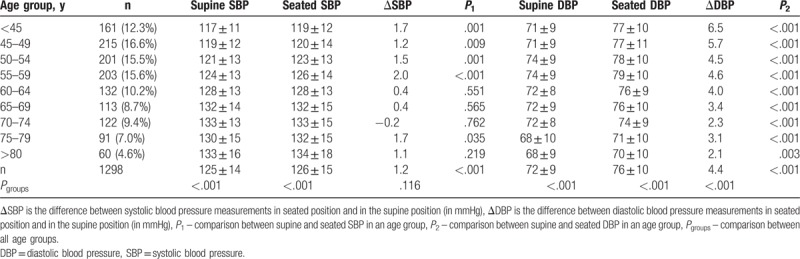
Comparison between supine and seated blood pressure (mm Hg) in different body positions and age groups.

### 3.3 Clinical and epidemiological implications in the diagnosis of hypertension

**Figure 5:**
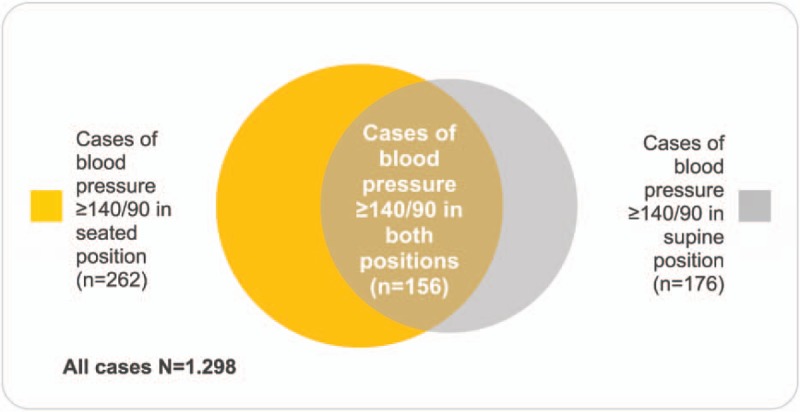
Comparison of high blood pressure prevalence using either of the methods of measuring the blood pressure.

We could identify 262 subjects with blood pressure above the cut off value of 140/90 mm Hg in the seated position and 176 subjects in the supine position. In 156 subjects, blood pressures of ≥140/90 mm Hg were found in both positions. The occurrence of blood pressures of ≥140/90 mm Hg was significantly higher in seated position than in the supine (19.9% vs 13.5%, *P* < .001, Chi-square = 20.3) (Fig. 5).

### 3.4 Factors associated with positional blood pressure difference

Linear regression models were used to investigate factors associated with positional difference in SBP and DBP. In the first regression model, sex, age, BMI, and diabetes mellitus status were used as independent variables and ΔDBP as the dependent variable. Age (β = −0.105, *P* < .001) and diabetes mellitus (β = −1.370, *P* = .012) were inversely associated with mean ΔDBP, whereas BMI was positively associated with ΔDBP (β = 0.160, *P* < .001). Sex did not show any significant association. In a second model, the ΔSBP was used as a dependent variable and the same variables as previously were used as the independent variables. In this model, none of the above variables were significantly associated with ΔSBP (Table 4).

**Table 4:**
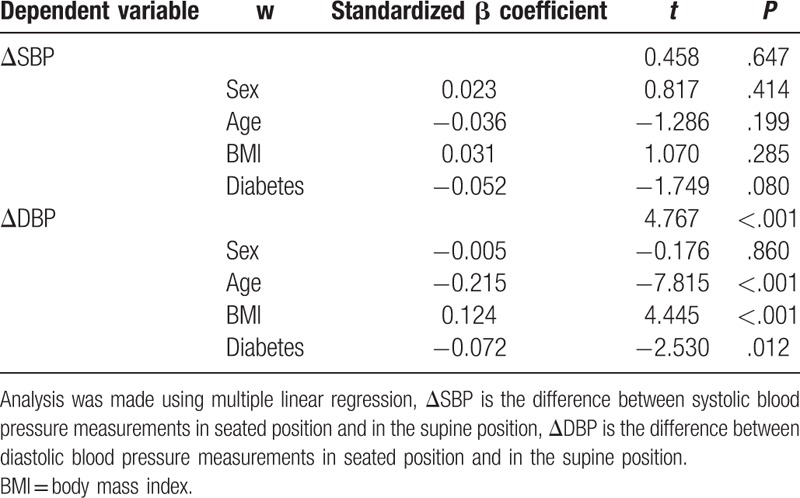
Analysis of factors associated with the postural change in blood pressure measurements.

## 4 Discussion

### 4.1 Principal findings

This population-based study showed that both men and women had significantly higher seated SBP and DBP. Moreover, the study showed that these postural differences were larger in the measurement of DBP and declined significantly by age. These differences resulted in a notably higher occurrence of high blood pressure if measured in the seated position. Comparing these results to previous work in this area, some major differences were found. Most of the studies after 2003 show higher blood pressure in the supine position. T-M et al^[8]^ in a large cohort of 6485 healthy subjects found that the supine SBP and DBP were 4.2 and 1.1 mm Hg higher than seated (*P* < .001). Others, such as Sala et al^[9]^ and Lu et al^[5]^ have also reported higher supine blood pressure readings, with their study cohorts consisting entirely of hypertensive patients. Our findings support the study from Krzesinski et al,^[10]^ who, on average, also found higher blood pressure readings in the seated position (5.7 mm Hg for SBP and 3.0 mm Hg for DBP, *P* < .001). Lacruz et al^[11]^ presented similar results in a paper from 2017, showing higher seated DBP in both sexes, while men also had higher seated SBP. In our study, the same results were found for both sexes. One possible explanation for the differences in the results across studies could be selection of the study population. However, a different study population structure might not play a crucial role. Studies that used cohorts of only young healthy subjects or only elderly with hypertension^[5,8]^ did not show a significant impact of the selected study population on reported results. Next, the sequence of blood pressure measurements could potentially contribute to the divergence in results. It has been shown that the sequence of blood pressure measurements can have a significant effect on the blood pressure, with a higher seated SBP and DBP when the sequence seated-supine is used.^[19]^ In our study as in other with results similar to ours,^[11]^ the seated measurement was first done, followed by the supine. In a contrast, both studies that showed different results compared with ours^[5,8]^ measured blood pressure first in supine position, followed by a seated position. No detail on the sequence were described in the work of Krzesinski et al.^[10]^ Although it was reported, that the sequence of measurements does not cause any significant carry-over effect on the postural difference,^[19]^ a randomized sequence of measurements would be the optimal choice to investigate the role of the posture. The number of measurements could also have an important effect; a low number of blood pressure readings leads to a higher error, whereas high number of readings extends the time the patient spends relaxing, which has shown to produce lower blood pressure results.^[20]^ The diagnostic value of supine blood pressure is unclear. We noted a much lower prevalence of high blood pressure in supine position, which means that measuring blood pressure only in supine position misses a significant number of actual hypertensive patients. However, Krzesinski et al^[10]^ demonstrated that the cut-off value of supine blood pressure ≥130/80 mm Hg was more precise than seated blood pressure ≥140/90 mm Hg in the diagnosis of hypertension, as it identified patients with increased night-time blood pressure better. Still, they concluded that the in-office seated blood pressure value ≥140/90 mm Hg remains more accurate with elevated daytime blood pressure. Trend analyses revealed a significant negative association between increasing age and positional difference in DBP, but not in SBP. To further explain the association and reveal possible confounding, we used several different multiple linear regression models. We observed that age, BMI, and diabetes mellitus shared a strong association with difference in DBP between the 2 positions. In fact, age was inversely associated with postural difference in DBP, while BMI was positively associated with postural differences. Subjects with diabetes had significantly lower postural differences than subjects without diabetes regardless of age. In contrast to other studies,^[5]^ sex did not show any significant association. Several factors might explain these associations. In fact, aging and diabetes are strongly correlated with cardiovascular medication^[21]^ that influences the autonomic nervous system. This could cause the loss of the adaptation of the circulation during the postural change and thus lower postural differences. To explore the possible confounding effect of cardiovascular medication, we adjusted our analysis using known hypertension as a proxy variable, as these patients usually have prescribed cardiovascular medication. However, the adjustment had no significant effect on the regression models and trend analysis. Similarly, Lu et al^[5]^ reported that neither monotherapy or combination drug therapy predicted posture-related changes in SBP and DBP. The influence on autonomic regulation of blood pressure can also be directly exerted by the age and diabetes^[22]^ and linked to obesity as well.^[23,24]^ However, with BMI being associated with higher postural difference, this might suggest a different type of autonomic dysregulation in obese people or another underlying mechanism. Such an alternative mechanism could be the difference in arterial stiffness. The relationship between age and increased arterial stiffness is well documented,^[25]^ while its relation with BMI is ambiguous.^[26]^ An increased cardiac output and hypervolemia have been described in patient with obesity. Combined with a stiffer arterial bed, this could explain the increase in postural change with increasing BMI.^[27]^ Finally, in concordance with previous observations, women had significantly lower both SBP and DBP, regardless of the body posture; however, the position-related differences in blood pressure were similar to those in men.

### 4.2 Strengths and weaknesses of the study

This study had a well-characterized large sample size with a high participation rate and was representative for a Caucasian population. The inclusion of a large range of ages permitted the investigation of the age differences in the postural changes of blood pressure measurements. One of the weaknesses of our study is the nonrandomized order of blood pressure measurements. In addition, the number of blood pressure measurements for each position was low (only 2); however, on average, the variation between the 2 measurements was relatively small. The time between the positions, during which the pulse wave measurements were conducted, was not standardized, but the impact in our results should be minimal.

### 4.3 Meaning of the study

In conclusion, this study confirms and expands existing evidence that there is a significant difference in blood pressure between seated and supine position. Measuring blood pressure in the supine position shows lower blood pressure readings when compared with the seated position. The results reinforce the importance of following the international hypertension guidelines – blood pressure must be measured in the seated position to ensure a correct diagnosis. As supine blood pressure measurement was a standard practice 10 years ago with most Swedish primary care doctors, and the DBP in seated position is higher, the change in routines may have resulted in an increase in the prevalence of hypertension. Clinicians should be aware of how age, BMI, and diabetes influence these differences. Future research to elucidate the mechanisms behind these differences is warranted.

## Acknowledgment

We are very thankful to every participant from Vara and Skövde whose contribution made our study possible.

## Author contributions

**Conceptualization:** Ernest Privšek, Margareta Hellgren, Lennart Råstam, Ulf Lindblad, Bledar Daka.

**Data curation:** Ulf Lindblad, Bledar Daka, Ernest Privšek

**Formal analysis:** Ernest Privšek, Bledar Daka, Ulf Lindblad

**Methodology:** Margareta Hellgren, Lennart Råstam, Ulf Lindblad, Bledar Daka.

**Resources:** Lennart Råstam, Ulf Lindblad, Bledar Daka, Margareta Hellgren

**Supervision:** Bledar Daka, Ulf Lindblad

**Validation:** Bledar Daka, Ulf Lindblad

**Visualization:** Ernest Privšek.

**Writing – original draft:** Ernest Privšek, Ulf Lindblad, Bledar Daka.

**Writing – review & editing:** Ernest Privšek, Bledar Daka, Ulf Lindblad, Margareta Hellgren, Lennart Råstam
